# Evaluation of Mechanical Properties of a Hollow Endodontic Post by Three Point Test and SEM Analysis: A Pilot Study

**DOI:** 10.3390/ma12121983

**Published:** 2019-06-20

**Authors:** Giuseppe Lo Giudice, Edoardo Ferrari Cagidiaco, Roberto Lo Giudice, Francesco Puleio, Fabiana Nicita, Massimo Calapaj

**Affiliations:** 1 Department of Biomedical and Dental Sciences and Morphofunctional Imaging, Messina University, 98122 Messina, Italy; logiudiceg@unime.it (G.L.G.); francesco.puleio@live.it (F.P.); fabin92@hotmail.it (F.N.); calapaj.massimo@alice.it (M.C.); 2 Department of Prosthodontic and Dental Materials, Siena University, 53100 Siena, Italy; edoardo.ferrari.cagidiaco@gmail.com; 3 Department of Clinical and Experimental Medicine; Messina University, 98122 Messina, Italy

**Keywords:** endodontic post, flexural strength, Young’s modulus, three-point bending test

## Abstract

The aim of this study was to investigate the mechanical properties of a fiber hollow endodontic post characterized by the presence of an empty central cylindrical channel extended along the whole length. This particular shape allows clinicians to use the post also as a cementation resin carrier. Ten hollow posts were divided in two groups: the control group (unfilled hollow posts) (Group 0) and hollow posts filled with dual resin cement (Group 1). The samples of both groups were subjected to mechanical and micromorphological analysis by performing a three-point test and SEM observations. In the three-point test, the Group 1 samples exhibited a fracture load of 57.09 ± 5.06 N, a flexural strength of 1323.53 ± 110.09 MPa, and a Young’s modulus of 42.87 ± 0.86 GPa. The samples of Group 2 exhibited a fracture load of 38.17 ± 1.7 N, a flexural strength of 908.87 ± 30.98 MPa, and a Young’s modulus of 40.33 ± 1.9 GPa. The difference between fracture load, flexural strength, and deflection between the two groups was statistically highly significant (*p* < 0.01). Further, the difference between the Young’s modulus of the two groups was statistically significant (*p* < 0.05). The values obtained are similar to those of other posts available on the market.

## 1. Introduction

Nowadays in post-endodontic restorations the gold standard for the replacement of tooth tissues is represented by composite posts, thanks to their mechanical properties more similar to dentin than old metal pin [1].

Several authors have stated that the use of posts does not provide any improvement of the tooth resistance, while it may represent a potential cause of root fracture in unfavorable anatomic situations or when the distribution of occlusal forces increases the compressive stress on the post-space dentinal surface [2,3].

The choice to use a post in order to improve the retention of post endodontic restorations is still debated. Although many authors considered that the presence of a post improves core retention, it is not possible to standardize the indication according to the number of residual walls or other reference parameters [4].

A recent review proved that it is not clear if endodontic posts are necessary even in cavities without residual walls, both in direct and indirect restorations [5]. Moreover, Naumann et al. statedthat the number of residual cavity walls is a crucial factor for the survival of post-endodontic restorations [6].

Detachment, one of the main causes of restoration failure with fiber posts, could be linked to different variables such as root canal cleaning and adhesion techniques, dentinal interface, polymerization shrinkage and the difficulty of photo induction in the deepest areas of the post space [7,8,9,10,11,12], along with the possible formation and dislocation of air bubbles in the apical part of the post space during the cementation steps [13]. 

The transversal incidental forces on the restoration determine stresses on the coronal and radicular dental tissues and along the post; thus it is essential that the shape and structure of the post will promote a uniform distribution of the traction and compression vectors, avoiding excessive loads and consequent dentinal microcracks [14,15,16,17]. Consequently, the mechanical properties of the endodontic posts have become the subject of different evaluations in order to describe the behavior of the material subjected to static and dynamic forces [1,18,19,20].

Calapaj et al. highlighted the clinical efficiency of a post-endodontic restoration system that used a metallic carrier for the resin cement injection, which remained incorporated after the polymerization phase [21]. The evolution of this technique led to the use of a carrier that is not metallic but made with silica fibers reinforced with composite resin in order to improve the flexibility and prevent excessive stresses on the root canal walls.

The aim of our research is to describe the characteristics of this new carrier/post in order to perform post endodontic restoration. The study compares the mechanical proprieties between empty and resin filled hollow posts. The null hypotheses considered no differences in mechanical behavior between filled or unfilled hollow posts. The alternative hypotheses considered that the post mechanical characteristics changed after filling.

## 2. Materials and Methods 

Our research was carried out on 10 hollow endodontic posts (Techole, Isasan, Rovello Porro, Italy) made of epoxy resin and reinforced with silica microfibers of cylindrical-conical shape with rounded tip and a diameter of 1.2 mm in the cylindrical portion. The structure is characterized by the presence, for more than 60% of the volume, of tensioned silica fibers parallel to the longitudinal axis of the post.

**Figure 1:**
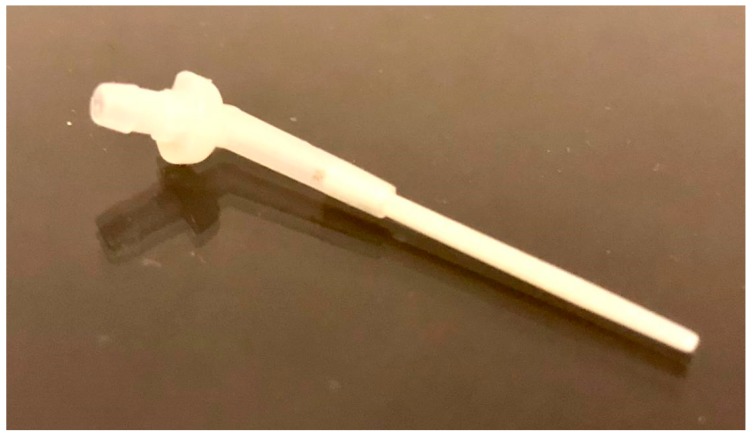
The tested hollow post.

The posts are characterized by an empty central cylindrical canal extended along the whole length. This distinctive shape allows the use of the post also as carrier for the cementation resin (Figure 1).

The posts were randomly divided into two groups (group 0 and group 1-# 5): five carrier-posts (Group 1) were connected with a dual cement syringe and filled with a single injection of dual curing composite cement (Clearfil DC Core Plus, Kuraray Noritake Dental, Tokyo, Japan). After the cement self-polymerization phase (3′), the posts were exposed to a curing light for 40″ (Valo, Ultradent Products, Inc. UT, USA). 

The group 0 hollow posts were not filled and used as the control group.

The sample size was established considering two independent groups (filled and unfilled posts) and as the primary endpoint the Young’s modulus. Assuming a two-sided significance level of 0.05, an expected mean for filled posts equal to 42 with an Standard Deviation (SD) of 10 (as the literature reported for posts with a filled structure) and an expected mean for unfilled posts equal to 24 (the lowest value found in literature for the Young’s modulus), it was determined that approximately five samples per group would be needed in order to guarantee a power level of 80% [1,18,21,22,23,24]. 

The samples of both groups were subjected to a mechanical and micromorphological analysis by performing a three-point test and SEM observations (Phenom Pro 5, Phenom-World B.V., Eindhoven, The Netherlands).

The three-point test was performed with an electronic dynamometer (Triax 50, Controls, Milan, Italy) at the Department of Medical Biotechnologies of the University of Siena.

The test, performed according to the ISO 14125 protocol, was carried out applying to the posts a bending force and recording the following parameters: fracture load, flexural strength, deformations and Young’s modulus.

To perform the three-point test, carried out at room temperature and humidity, the samples were placed on two high speed steel rollers with a diameter of 2 mm, parallel to each other at a distance of 13 mm; the load was exerted with gradual progression at the centerline point at a speed of 1 mm/min

The deformation of the samples was recorded until the break and was correlated with the loads exerted.

The flexural strength (δ*_f_*) and the modulus of elasticity (E*_f_*) were calculated using the following formulae:

(1) $$E=\frac{S4_{max}l^{3}}{3\pi d^{4}}\;\delta_{f}=\frac{8F_{max}l}{\pi d^{3}}$$

where *F_max_* represents the maximum applied load (N), *l* is the distance between the two supports (mm), *d* is the diameter of the samples (mm), *S* is the ratio between applied force (N) and deformation (mm).

The statistical significance between the average values identified in the two groups was evaluated by the Student’s t-test with a 5% increased confidence level considering the small size of the sample. The value was obtained using SPSS 17.0 for the Windows operating system.

After the three-point test had been performed, the surface of all samples was observed by SEM at the resolution of 3072 × 2304 pixels, with a magnification of 50×, 250×, 1000×, in order to analyze the post fracture line of both groups.

## 3. Results

The average value of each experimental parameter and the standard deviation obtained in the mechanical tests are shown in Table 1.

The group 1 three-point test, reached the yield point when loaded with an average force of 57.09 ± 5.06 N (Min 53.75 N Max 65.49 N), a flexural strength of 1323.53 ± 110.09 MPa (Min 1233.18 MPa, Max 1502.63 MPa) with an average deflection of 0.77 ± 0.07 mm (Min 0.72 mm Max 0.90 mm). The recorded Young’s modulus was 42.87 ± 0.86 GPa (Min 41.63 GPa, Max 43.81 GPa).

The group 0 (hollow posts) three point test reached the yield point when loaded with an average force of 38.17 ± 1.7 N (Min 36.39 N, Max 40.66 N), a flexural strength of 908.87 ± 30.98 MPa (Min 880.84 MPa, Min 958.07 MPa) with an average deflection of 0.57 ± 0.03 mm (Min 0.52 mm, Max 0.60 mm). The recorded Young’s modulus was 40.33 ± 1.9 GPa (Min 38.43 GPa, Max 43.28 GPa).

The difference between fracture load, flexural strength, and deflection between the two groups was statistically highly significant (*p* < 0.01). The difference between the Young’s modulus of the two groups was also statistically significant (*p* < 0.05).

**Figure 2:**
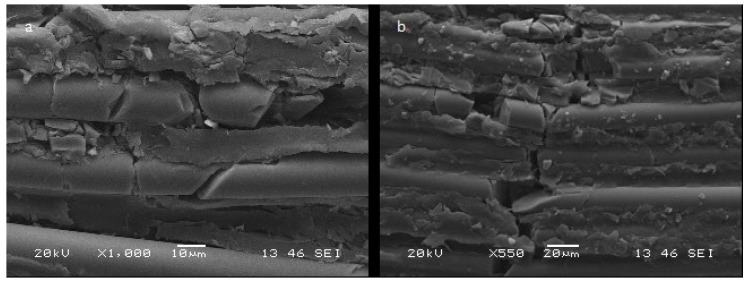
Fractures of the reinforcing fibers and of the resin matrix. (**a**) Group 0, (**b**) Group1.

The images obtained by Scanning Electron Microscope (SEM) show surface linear fractures, resin matrix crack, and failure of the reinforcing fibers in both experimental groups. The fibers appear fractured in several points (Figure 2). 

The observations within the sample of same experimental group seem substantially similar.

**Figure 3:**
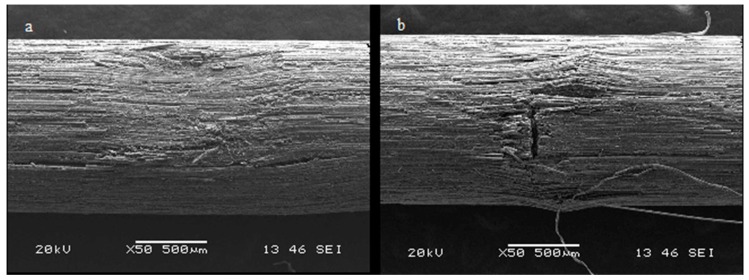
Impression of the dynamometer tip on the surface of the post. (**a**) Group 0, (**b**) Group1.

In all the posts, the impression of the dynamometer tip is visible at the point of application (Figure 3).

**Figure 4:**
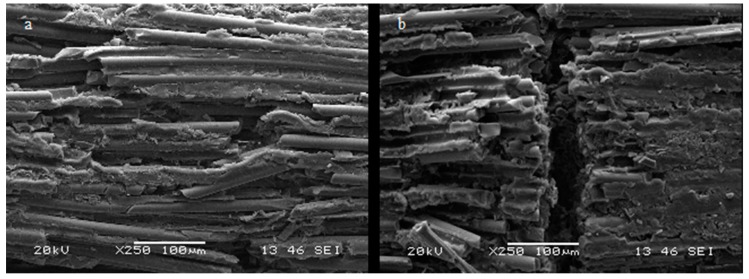
(**a**) Group 1: fracture line: oblique direction of fracture rim, (**b**) Group 0: the fracture direction is perpendicular to the post long axis.

The direction of the fracture line in the Group 1 samples is mainly oblique (Figure 4a).

In the Group 0 samples, the fracture direction is perpendicular to the post long axis (Figure 4b).

## 4. Discussion

Post mechanical characteristics should be evaluated when considering the performance in relation to the occlusal forces. The static analysis, like the three-point test, involves the application of an increasing unidirectional force on the sample over time; the maximum fiber stress at failure is recorded with the deformation as the applied force increases. From this test it is possible to extrapolate fracture load (N), flexural strength (MPa), Young’s modulus (GPa), and maximum deflection (mm). Fracture load and flexural strength could be defined as the values when it the material deformation type from plastic to elastic is noticeable and therefore constitutes an irreversible deformation. Young’s modulus, in the case of uniaxial load conditions, is the ratio between the tension and deformation of an elastic material.

A comparative evaluation of the experimental results shows that the elasticity values obtained in the group of hollow posts filled with dual cement are similar to fiber posts with a comparable composition available on the market [18,21,22,23].

Numerous studies have examined the Young’s modulus of the glass fiber posts elasticity (glass, quartz and silica). We can state that the posts tested have, when filled, an elasticity near to the highest values reported in the literature [1,18,21,22,23,24].

Considering the distinctive structure of the analyzed posts, it is also necessary to evaluate the elastic behavior of the unfilled hollow post in order to identify an overall assessment of the endocanalar retention system.

The mean value of elasticity recorded in the unfilled hollow posts was lower than the values recorded for the filled posts, and within the range of elasticity identified for silica fiber posts [1,22,23,24]. The statistical analysis shows highly significant differences. Therefore, the null hypothesis is rejected, and it can be stated that the posts filling determines a statistically significant difference of elasticity between filled and unfilled hollow posts.

Analyzing the values of fracture load, flexural strength, and maximum deflection, it is evident how the filling of the fiber carrier leads to a substantial improvement of performance.

After being filled with cement, the flexural strength of the hollow post increased and reached the higher values for posts with similar reinforcing fibers [1,22,23,24].

Comparing the hollow and not hollow posts with similar fiber composition, for the parameter “flexural strength” the hollow post shows higher values than classic posts with the same or higher diameter [1,22,23,24,25].

Many Authors have asserted that the post should have a Young’s modulus similar to the dentin one (18–25 GPa) in order to obtain structural coherence and a better distribution of forces [26,27,28]. The modulus of elasticity of the tested post is higher than the modulus of elasticity of the dentin.

However, this value does not appear to be linked to a substantial decrease in root resistance to stresses. In fact, many researches analyzing root fractures of teeth restored using metal posts have stated that the failures are due to the excessive stiffness of the metal and not related to the different elasticity [29]. However, more recent research has reported an absence of statistical significance in the root fractures percentage when comparing different posts stiffness [30,31,32].

Post length, taper and diameter are important parameters for the survival rate of post-endodontic restorations [33].

Images obtained by SEM scans show linear fractures of reinforcing fibers and resin matrix in both experimental situations. (Figure 2).

In all posts, the impression of the point of application of the dynamometer tip is visible (Figure 3).

The fracture line in the Group 1 samples has an oblique direction related to the major axis of the post (Figure 4a). The progress of the fracture line in the Group 0 samples is predominantly perpendicular. (Figure 4b). 

The differences on the fracture line direction among the groups, recorded after the three point bending test, is linked to a different forces distribution and could be compared, in the engineering field, to the deformation of empty and hollow pipes. The applied force determines flexion and roundness of the hollow post until fiber and resin matrix crack that occur perpendicular to the post long axis. 

The presence of resin filling modifies the fracture dynamics reducing the roundness and due to the factorization of the vector forces applied during the three-point test results in an oblique fracture line [34,35,36]. 

The analysis of fracture line direction confirm that the filling modifies the post’s structural behavior. 

## 5. Conclusions

The mechanical properties of the tested posts are effective if compared to other posts with a similar composition [1,22,23,24]. This conclusion could be applied to both filled and non-filled hollow posts, although obviously the performance of filled hollow posts is better.

The technique that involves the use of a post as carrier simplifies the operative protocol, allowing placement in the post space and filling with composite cement at the same time.

The retention system thus obtained is more resistant than a normal post with the same diameter thanks to a different distribution of forces along the post.

Considering this study as a benchmark and the small sample studied, further studies are necessary in order to evaluate the clinical advantage and disadvantage of this system. Moreover, further research could be useful to evaluate the modification of the post’s mechanical characteristics in relation to different post diameters and resin cement characteristics. 

## Figures and Tables

**Table 1:** Average value and standard deviation obtained in three-point test.

Groups	Fracture Load (N)	Flexural Strength (MPa)	Maximum Deflection (mm)	Young’s Modulus (GPa)
Group 0	38.17 ± 1.7	908.87 ± 30.98	0.57 ± 0.03	40.33 ± 1.9
Group 1	57.09 ± 5.06	1323.53 ± 110.09	0.77 ± 0.07	42.87 ± 0.86
